# A Retrospective Evaluation of the Utility of Capsule Endoscopy and Double-Balloon Endoscopy in Crohn's Disease

**DOI:** 10.1155/2016/1085027

**Published:** 2015-12-30

**Authors:** Masanao Nakamura, Yoshiki Hirooka, Osamu Watanabe, Takeshi Yamamura, Kohei Funasaka, Eizaburo Ohno, Hiroki Kawashima, Ryoji Miyahara, Hidemi Goto

**Affiliations:** ^1^Department of Gastroenterology and Hepatology, Nagoya University Graduate School of Medicine, 65 Tsurumai-cho, Showa-ku, Nagoya, Aichi 461-0031, Japan; ^2^Department of Endoscopy, Nagoya University Hospital, 65 Tsurumai-cho, Showa-ku, Nagoya, Aichi 461-0031, Japan

## Abstract

*Background*. Although the usefulness of capsule endoscopy (CE) and double-balloon endoscopy (DBE) for the evaluation of Crohn's disease (CD) is established, their capabilities in the differential diagnosis of small bowel stenosis have not been sufficiently addressed. The present study therefore aimed to retrospectively determine the types of patients for whom CE and DBE would confer the most benefit. *Patients and Methods*. We retrospectively reviewed data from 185 patients with established CD. A change of treatment based on CE or DBE results or successful DBE balloon dilation was defined as clinically useful indication. We then analyzed the factors significantly related to useful and poor indications. *Results*. CE results were assessed as useful indications in 28 (45%) of 62 patients. Multivariate analysis demonstrated that positive CRP and low IOIBD score are factors significantly related to a useful indication. DBE results were recognized as useful indications in 118 (77%) of 153 patients. Multivariate analysis indicated small bowel stenosis and abdominal pain as factors significantly associated with useful indications. All patients with a poor indication on CE had small bowel stenosis. *Conclusions*. CE was most useful for patients in clinical remission with positive CRP and without stenosis, whereas DBE was useful for patients with symptoms of stenosis.

## 1. Introduction

Crohn's disease (CD) is a progressive disease associated with a high risk of complications, including strictures, fistulae, perianal complications, and colorectal cancer, over time [1]. CD patients are at high risk of requiring surgical procedures over time due to the development of complications, such as strictures. Capsule endoscopy (CE) and double-balloon endoscopy (DBE) are methods used to evaluate small bowel lesions. These methods are also capable of evaluating small bowel stenosis by optical observation, whether it is inflammatory or fibrotic. However, the use of these modalities to differentially diagnose CD with small bowel stenosis or to assess therapeutic effects has not been sufficiently investigated.

CE and DBE are considered complementary with regard to diagnosing small bowel disease, and each method has unique characteristics [2, 3]. CE is less invasive for patients and is capable of exploring the whole small bowel in a single examination. It can also be utilized to monitor disease activity, evaluate therapeutic response, and detect postoperative recurrence in established CD [4]. To avoid CE retention, a patency capsule is utilized prior to CE. The performance of CE in diagnosing CD can be superior to that of other modalities [5–8]. On the other hand, DBE is useful for detailed observation, obtaining small bowel biopsies, and balloon dilation to relieve small bowel stenosis [9, 10]. Subsequent histopathological examination can provide beneficial information to aid in assessing the severity of inflammatory changes. DBE can be utilized to identify suitable CD treatment strategies in otherwise inconclusive cases. However, indications for DBE should be carefully considered because it is more invasive than CE and is associated with a risk of gastrointestinal perforation [11].

Despite the increasing use of immunosuppressive antitumor necrosis factor treatments and elemental diets, approximately half of CD patients still require surgery within 10 years of diagnosis [12]. In particular, small bowel stenosis is the main reason for scheduling surgery [12–14], and symptoms of stenosis are a major concern because they impair the quality of life of patients with CD.

The use of CE or DBE may be very important so that inflammatory activity in patients with small bowel stenosis can be precisely evaluated at the pretreatment step. Few studies have evaluated small bowel lesions on CD images with a focus on stenosis. One reason for this lack of study might be that the endoscopic evaluation of small bowel stenosis and balloon dilation are performed by selected experts' hands [15, 16]. The roles for CE and DBE in the assessment and treatment of established CD, including the management of small bowel stenosis, still need to be defined [17].

The present study aimed to retrospectively determine which patients with established CD would benefit from an assessment by CE and DBE and to identify the roles of both modalities in the management of CD, including the issue of small bowel stenosis.

## 2. Patients and Methods

We analyzed data from 185 patients with established CD who underwent CE, DBE, or both methods as the initial examination at our hospital between June 2003 and August 2014. A PillCam patency capsule was routinely used prior to CE to confirm the patency of the gastrointestinal tract. Although a PillCam patency capsule was administered in 80 patients, 62 patients (77.5%) had patency of the GI tract. Thirty-two, 123, and 30 patients were examined by CE alone, DBE alone, and both modalities, respectively. CE was the initial procedure for all of the patients who had both modalities. The mean age of the patients was 38 years, and most patients were male. A history of surgery, symptoms, and clinical activity was recognized in 46%, 76%, and 35% of the patients, respectively.

The CE results were classified into 3 categories, “Good,” “Moderate,” or “Poor,” based on their contribution to the evaluation of the patient (Table 1). For example, in cases that treatment was implemented based on the discovery of an active lesion on CE images, CE was considered to have played an important role as a good indication. If CE confirmed mucosal healing and the physician did not modify the treatment, the role of CE was defined as a moderate indication.

**Figure 1:**
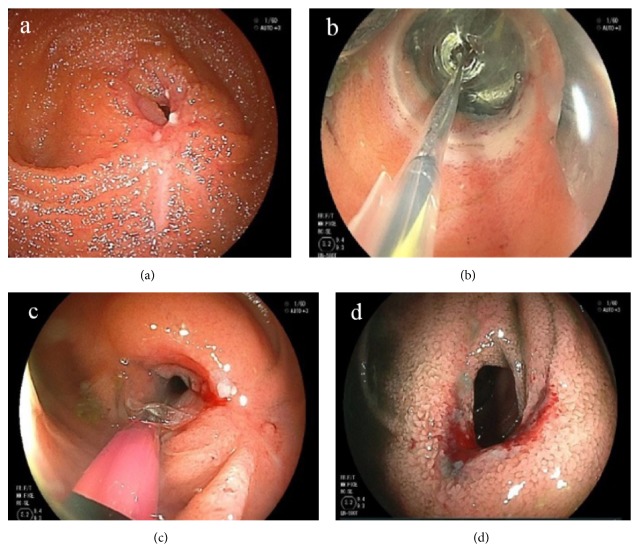
(a) Fibrotic stenosis in the small bowel. (b) Balloon dilation using double-balloon endoscopy. (c) Just after dilation. A small amount of bleeding was the sign of successful dilation. (d) Dilated lumen.

The results of DBE were also categorized into “Good,” “Moderate,” or “Poor” based on their clinical contribution (Table 1). For example, when treatment was modified based on the results of DBE or when balloon dilation was successful, DBE was considered to have played an important role (Figures 1(a)–1(d)).

The primary endpoint of this retrospective study was the background of the CD patients in whom CE or DBE had an important role in facilitating the management of CD with small bowel stenosis. The secondary endpoints were the comparison of the clinical findings between patients of different backgrounds at the time of the first CE and DBE and the poor indications for using these modalities to evaluate small bowel stenosis.

### 2.1. Statistical Analysis

The statistical software package SPSS for Windows (SPSS, Chicago, IL, USA) was used to analyze the data. To compare the backgrounds of the patients who underwent CE and/or DBE, a Mann-Whitney test was used. Univariate and multivariate logistic regression analyses were used to identify the factors related to good and poor indications for CE and DBE. In all analyses, a *P* value of less than 0.05 was considered statistically significant.

## 3. Results

Table 2 shows a comparison of backgrounds between patients who underwent CE versus DBE examination first. The 30 patients who underwent both examinations were included in each group. The DBE group involved significantly more male patients with symptomatic disease, high clinical activity, and small bowel stenosis.

The categories “Good,” “Moderate,” and “Poor” based on the CE results were noted for 28, 24, and 10 of the 62 patients, respectively. Factors related to the “Good” rating were investigated using univariate and multivariate analyses. Both approaches demonstrated that positive CRP and IOIBD score of 0 or 1 were significant factors playing a good role in assessing the effects of treatment and the status of the patient (Tables 3 and 4). On the other hand, all of the patients for whom the CE result was in the category “Poor” had small bowel stenosis. We then investigated background factors that affected the category “Poor” in patients with stenosis. Patients with small bowel stenosis who underwent CE had more clinical activity and history of surgery than those without stenosis (Table 5).

The DBE results were categorized into “Good” in 118 (77%) of 153 patients, indicating the usefulness of DBE as a management tool. The other results were considered “Moderate” in 28 patients and “Poor” in 7. Univariate analysis presented small bowel stenosis and abdominal pain as background factors which were good indications for DBE (Table 6). These factors and the patients' history of surgery were evaluated by multivariate analysis. The factors small bowel stenosis and abdominal pain were selected as significant background factors for DBE that play a favorable role in discerning the effects of treatment (Table 7).

All 7 patients with the category “Poor” in the DBE group had small bowel stenosis. We subsequently investigated which background factors affected the poor indication among the patients with small bowel stenosis. A history of surgery tended to be related, but no single factor was significantly involved (Table 8).

## 4. Discussion

It can take a long time for symptoms to develop after the formation of small bowel lesions in CD, which often complicates early diagnosis [18]. Accordingly, patients are sometimes diagnosed with longitudinal ulcers, stenosis, or fistulae at the time of their first visit. The evaluation for these patients is likely to be limited by a narrow lumen, the lesion variety, and abdominal adhesion after surgery. Therefore, the small bowel should be evaluated by endoscopic modality at the early stage of CD development. Endoscopy also plays an essential role in the evaluation and monitoring of established Crohn's disease [19]. In the present study, we assessed the roles of CE and DBE with regard to evaluating the status of the mucosa and therapeutic effects in patients with small bowel stenosis.

**Figure 2:**
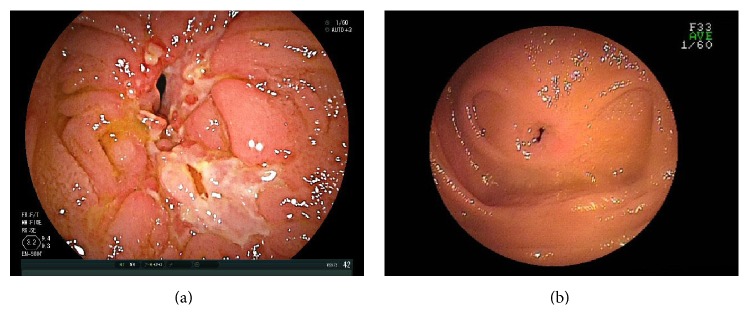
(a) Small bowel stenosis containing both fibrotic and inflammatory components. (b) Small bowel stenosis with fibrosis.

A good indication for CE was positive CRP, even when patients were asymptomatic in clinical remission (Table 7). In that case, active small bowel lesions can be discovered using CE, and such findings can influence decisions to change and/or reinforce treatment. Early treatment reinforcement is desirable because the treatment of jejunal lesions discovered by CE is sometimes challenging [20–22]. Because elevated CRP often correlates with CD activity [23–26], CE should be suggested when the cause of such an elevation is unknown. DBE frequently plays a role in differentially diagnosing patients with abdominal pain and abnormal borborygmus induced by severe small bowel stenosis (Table 9). Whether such stenosis is fibrous or edematous accompanied by active ulcers is often indeterminable by the imaging modalities because bowel thickness on images can reflect both the presence of an inflammatory component and fibrosis in deep layers. Because the stenotic region can be directly observed using DBE, active lesions on the surface of the stenosis and stenotic rigidity can be analyzed (Figures 2(a) and 2(b)). Furthermore, balloon dilation can be applied to fibrous stenosis in order to avoid surgical treatment.

On the other hand, CE and DBE could not play roles in the management of small bowel stenosis for certain patients. CE can be safely applied after confirming the patency of the GI tract using a patency capsule [27, 28]. However, the results in Table 7 indicate that the status of small bowel stenosis can easily change when inflammatory activity is severe or the stenosis is very narrow due to an edematous component. Thus, CE should be carefully scheduled with optimal timing. Although DBE was effective for patients with small bowel stenosis, Table 8 shows a relation between poor indication for DBE and history of surgery. Patients with a history of surgery may still have active CD status according to poor response to medical treatment after surgery and have ulcers in stenotic regions. Thus, DBE should be indicated, considering a history of abdominal surgery and the status of inflammatory activity.

Recent studies have identified mucosal healing on endoscopy as a key prognostic parameter in the management of CD, thus highlighting the role of endoscopy in monitoring disease activity. In fact, mucosal healing has emerged as a key treatment goal in CD that predicts sustained clinical remission and resection-free survival of patients [29–33]. Thus, the small bowel mucosa must be periodically evaluated by CE and DBE, which are optimum modalities for confirming sustained remission based on mucosal healing. The physician does not modify the treatment of CD when mucosal healing and clinical remission are confirmed; however, this step is sometimes important for some types of patients whose bowel easily reactivates. Mucosal healing on scheduled follow-up endoscopy was reported to be significantly associated with a lower risk of colectomy in ulcerative colitis and a decreased rate of corticosteroid therapy in CD [34]. On the other hand, Kim et al. reported that the cumulative surgery rate was not significantly different between follow-up endoscopy and any of the follow-up groups in patients with CD, though the rate of management change was significantly higher in the group with indications for follow-up endoscopy [35]. Therefore, such follow-up examination should be conducted in a noninvasive and safe manner because it depends upon the patient's acceptance and will be performed at regular intervals. CE was more frequently used than DBE to confirm clinical remission in the category “Moderate” (28 (45%) of 62 versus 28 (18%) of 153 patients). CE may be appropriate to periodically confirm that treatment is satisfactory for patients in remission.

This study has some limitations due to those inherent in a single center and retrospective design, and the backgrounds of the patients had bias between the CE and DBE groups. Inflammatory activity was significantly higher in the DBE group. However, this background might be significant because it matches the real clinical situation. Because a high proportion of patients develop small bowel stenosis over the course of CD and it affects the cumulative risk of hospitalization, further prospective study will be requested.

In conclusion, CE was useful for patients in clinical remission with elevated CRP and no small bowel stenosis, whereas DBE was useful for patients with abdominal symptoms due to small bowel stenosis, even though inflammatory activity was low.

## Figures and Tables

**Table 1:** Categorizations of CE and DBE results.

Category	Details of CE results	Details of DBE results
Good	Treatment was changed by the CE result	Treatment was changed by the result Balloon dilation was successfully performed for small bowel stenosis

Moderate	Treatment was evaluated to be appropriate by the CE result	Treatment is evaluated to be appropriate by DBE result

Poor	Patency was not confirmed or CE had any adverse event	Not accessible to the small bowel or DBE had any adverse event

**Table 2:** Demographic data of the patients at the first examinations.

	CE	DBE	*P* value
*N*	62	153	
Age (y.)	37 ± 12	38 ± 12	0.5734
Gender (M/F)	23/39	115/38	<0.0001
BMI	20.4 ± 2.7	20.2 ± 2.6	0.2245
Symptom	18/62 (29%)	133/153 (87%)	<0.0001
History of surgery	41/62 (64%)	84/153 (55%)	0.1279
IOIBD score (0, 1)	50/62 (80%)	58/153 (38%)	<0.0001
CRP positive	19/62 (30%)	61/153 (40%)	0.2049
Small bowel stenosis	23/62 (37%)	128/153 (83%)	<0.0001

**Table 3:** Factors affecting the role of CE—univariate analysis.

Factor	Odds ratio	*P* value	(95% CI)
Age	0.984	0.5450	(0.934–1.041)
Gender	2.227	0.2568	(0.556–9.433)
BMI	1.221	0.1333	(0.945–1.567)
Stenosis on image	0.944	0.9446	(0.283–3.709)
CRP positive	18.989	0.0104	(1.981–180.72)
IOIBD score (0, 1)	18.564	0.0250	(1.434–237.756)
Symptom	1.167	0.8429	(0.241–5.505)
History of surgery	1.678	0.1823	(0.785–3.601)

**Table 4:** Factors affecting the role of CE—multivariate analysis.

Factor	Odds ratio	*P* value	(95% CI)
BMI	1.171	0.184	(0.927–1.477)
CRP positive	21.265	0.007	(2.399–193.357)
IOIBD score (0, 1)	16.856	0.022	(1.494–190.214)
History of surgery	1.611	0.182	(0.825–3.719)

**Table 5:** Comparisons between the groups with small bowel stenosis for affecting the category “Poor” on CE.

	Good and Moderate	Poor	*P* value
*N*	13	10	
Factor			
Mean age	36.5	43.7	0.1925
Gender (M/F)	3/13	3/10	0.7078
Mean BMI	20.5	19.7	0.3685
CRP ≧0.3 mg/dL; positive	5/13	4/10	0.9403
IOIBD score (0, 1)	12/13	4/10	0.0069
Symptom	3/13	6/10	0.0721
History of surgery	7/13	10/10	0.0191

**Table 6:** Factors affecting the role of DBE—univariate analysis.

Factor	Odds ratio	*P* value	(95% CI)
Age	1.011	0.5840	(0.972–1.052)
Female	0.894	0.8468	(0.283–2.800)
BMI	1.321	0.2124	(0.567–2.983)
Stenosis on image	15.267	<0.0001	(4.641–50.219)
CRP positive	0.901	0.8505	(0.306–2.655)
IOIBD score (0, 1)	0.980	0.9725	(0.313–3.069)
Symptom	5.399	0.0096	(1.508–19.308)
History of surgery	2.752	0.0808	(0.883–8.574)

**Table 7:** Factors affecting the role of DBE—multivariate analysis.

Factor	Odds ratio	*P* value	(95% CI)
Stenosis on image	14.848	<0.0001	(4.607–47.856)
Symptom	5.526	0.0052	(1.665–18.337)
History of surgery	2.630	0.0919	(0.856–8.100)

**Table 8:** Comparisons between the groups with small bowel stenosis for affecting the category “Poor” on DBE.

	Good and Moderate	Poor	*P* value
*N*	118	7	
Factor			
Mean age	38.3	39	0.8916
Gender (M/F)	91/27	5/2	0.7290
Mean BMI	20.9	19.1	0.3885
CRP ≧0.3 mg/dL; positive	47/118	5/7	0.1264
IOIBD score (0, 1)	45/118	4/7	0.4311
Symptom	108/118	6/7	0.4840
History of surgery	56/118	6/7	0.0617

**Table 9:** Indications of CE and DBE.

Modality	CE	DBE
Condition		
Abdominal pain	Poor	Good
Low clinical score	Good	Moderate
CRP positive	Good	Moderate
GI stenosis	Moderate	Good

## Abbreviations

CD: Crohn's disease
CE: Capsule endoscopy
DBE: Double-balloon endoscopy.
